# Trends in idiopathic inflammatory myopathies: cross-sectional data from the German National Database

**DOI:** 10.1007/s00296-020-04634-0

**Published:** 2020-06-27

**Authors:** Katinka Albrecht, Dörte Huscher, Johanna Callhoff, Jutta G. Richter, Tobias Alexander, Jörg Henes, Angela Zink

**Affiliations:** 1 German Rheumatism Research Centre, Epidemiology Unit, Charitéplatz 1, 10117 Berlin, Germany; 2 Charité Universitätsmedizin, Institute of Biostatistics and Clinical Epidemiology, Berlin, Germany; 3 Medical Faculty, Policlinic for Rheumatology and Hiller Research Centre for Rheumatology, Heinrich-Heine-University Duesseldorf, Duesseldorf, Germany; 4 Department of Rheumatology and Clinical Immunology, Charité Universitätsmedizin, Berlin, Germany; 5 Department of Rheumatology and Clinical Immunology, Eberhard Karls-University of Tuebingen, Tuebingen, Germany

**Keywords:** Idiopathic inflammatory myopathies, Polymyositis, Dermatomyositis, Patient-reported outcomes

## Abstract

**Objective:**

To describe trends in outcomes among patients with idiopathic inflammatory myopathies (IIM) over two decades.

**Methods:**

From 1997 to 2017, a total of 1079 IIM patients were documented in the National Database of the German Collaborative Arthritis Centers. Annual cross-sectional data on treatment, disease activity, patient-reported outcomes, hospitalization and employment were compared across the years. Information on phenotypes, organ manifestations and autoantibodies was collected for a subset to compare the assessment of global health, pain, fatigue and sleeping disorders.

**Results:**

In 2017, significantly more IIM patients were assessed to be in low disease activity (94%) than in 1997 (59%), *p* < 0.01. Pain (*p* = 0.001), global health (*p* = 0.049), fatigue (*p* = 0.03) and sleeping disorders (*p* = 0.01) also improved since recording. Glucocorticoid use decreased from 84 to 58% (*p* < 0.01). Employment in patients < 65 years remained unchanged (53%), while early retirement (23–9%), hospitalization/year (34–18%) and sick leave (52–24%) decreased. A total of 186 patients with information on subtypes were classified as polymyositis (44%), dermatomyositis (33%), anti-synthetase syndrome (10%), overlapping-myositis (8%), inclusion body myositis (2%), necrotizing myositis (0.5%) and unspecific (3%). The most frequently reported symptoms were limitations in global health (60%), fatigue (57%) and sleeping disorders (51%), and all of them were most frequent in overlap-myositis. Pulmonary hypertension and cardiomyopathy were associated with poor outcomes regarding global health, daily activities and fatigue.

**Conclusion:**

IIM patients report better outcomes than 20 years ago, along with good physician-reported disease control. Global health, fatigue and sleeping disorders are relevant patient-reported domains in IIM.

## Introduction

Idiopathic inflammatory myopathies (IIM) are a heterogeneous group of inflammatory muscle diseases that involve muscle weakness and visceral involvement, resulting in disability and impaired quality of life [1–3]. As IIM are rare among inflammatory rheumatic diseases, it is difficult to collect data for outcome research with a sufficient number of patients. In 2011, the Outcome Measures in Rheumatology (OMERACT) Myositis Special Interest Group was established to examine patient-reported outcome measures in myositis [4]. They required additional focus on patients with different myositis disease phenotypes and manifestations across a range of disease activity. Results from the OMERACT multicenter focus groups indicated that some of the patient-reported symptoms such as pain and fatigue have not been sufficiently assessed in myositis patients [5]. Due to the scarcity of patients with IMM, the data were often compiled or referred to small patient numbers [6, 7]. International collaborative research within the Euro Myositis registry enabled to increase patient numbers and therefore facilitates myositis research [8]. Until sufficient data are available, further efforts are made to focus on a core set of patient-reported domains, incorporating the patient perspective on the prioritization of outcomes [9, 10]. The modified patient-reported outcome core domain set includes muscle symptoms, fatigue, level of physical activity and pain as mandatory outcomes to assess in IIM [11]. In the German National Database (NDB) of the Collaborative Arthritis Centers, patient-reported outcomes have been documented for many years. The NDB was specifically conceptualized to provide data on health care for all patients with inflammatory rheumatic diseases. Although documentation is tailored to rheumatoid arthritis being the most frequently reported disease, data on spondyloarthritis, connective tissue diseases and vasculitis are also collected [12]. Patients with IIM have been observed since 1993. The aim of the present study was to describe trends in core domains such as fatigue, pain and other outcomes regarding work ability and hospitalization among patients with IIM over the last 20 years.

## Patients and methods

Cross-sectional data were derived from the National Database of the German Collaborative Arthritis Centers (NDB) between 1997 and 2017. The NDB is an ongoing prospective study which was established in 1993 as a long-term monitoring database for German rheumatology. Participating rheumatologists from 15 to 17 centers consecutively include unselected outpatients with inflammatory rheumatic diseases. Centers comprise both private practices and tertiary outpatient clinics. The database provides annually updated information on patients with inflammatory rheumatic diseases under rheumatologic care, covering data on sociodemographic parameters, laboratory tests, therapies, clinical and patient-reported outcomes, as well as additional health information [13]. Patients with IIM were identified by confirmed ICD-10 diagnosis of M33.0 (juvenile dermatomyositis), M33.1 (other dermatomyositis), M33.2 (polymyositis) or M33.9 (other dermatomyositis-polymyositis, not specified), International Classification of Diseases, 10th Edition. There was no patient in the NDB with main diagnosis of G72.4 (inflammatory myopathy, not elsewhere classified).

### Annual documentation

For each year, treatment is reported including the following substances: NSAIDs, glucocorticoids, azathioprine, methotrexate, cyclosporine A, cyclophosphamide, antimalarials, TNF inhibitors (since 2000), mycophenolate mofetil (since 2005), rituximab (since 2006). The glucocorticoid dose was categorized into < 7.5 and  ≥ 7.5 mg prednisolone equivalent per day. Disease activity is assessed by the rheumatologist on a numerical rating scale (NRS) with values ranging from 0 (no activity) to 10 (highest activity). Physicians also report on the presence of the following comorbid conditions: hypertension, cardiac disease, osteoporosis, osteoarthritis, diabetes, renal disease, depression and malignant neoplasm (yes/no). Utilization of physiotherapy within the last 12 months was reported by the patient until 2005 and from 2006 onward by the physician.

Patient-reported outcomes include pain, global health (since 2000), fatigue, physical activities, sleeping disorders, coping, emotional and physical well-being (all since 2007). All of them are assessed on NRS (0–10) that are derived from the rheumatoid arthritis impact of the disease (RAID) [14] and that are also used in other connective tissue diseases [12, 15]. Functional status is assessed by the Hannover Functional Ability Questionnaire (FFbH) ranging from 0 (no functional capacity) to 100 (full functional capacity) [16]. Anxiety/depression and daily activities (no/moderate/extreme) are assessed by the EuroQol 5D [17]. Patients were asked about their employment status (Are you full-time employed/part-time employed/unemployed/retired/in early retirement?), sick leave (Have you been on sick leave due to your inflammatory disease during the past 12 months?) and hospitalization (Have you been treated as an inpatient due to your inflammatory rheumatic disease during the last 12 months?).

### Myositis questionnaire

As information on IIM phenotype, myositis-specific autoantibodies and organ manifestations is not captured in the NDB, a supplementary questionnaire was sent out once in 2015. The rheumatologists were asked to complete the additional items for all 232 patients with IIM who had been documented between 2007 and 2015, covering data on the diagnostic subtype, in particular dermatomyositis (DM), polymyositis (PM), anti-synthetase syndrome (ASS), connective tissue disease—overlap, necrotizing myopathy and inclusion body myositis (assigned by the rheumatologist), confirmation of diagnosis (biopsy, magnetic resonance imaging, creatine kinase (CK), transaminase enzymes and/or antibodies), antibody tests and specification, if positive (Anti-Jo-1, anti-RNA, anti-Mi-2, anti-SRP, anti-HMG-CoA, anti-MDA-5 or other), history of organ involvement: skin, muscular, arthritis, dysphagia, interstitial lung disease, pulmonary hypertension, cardiomyopathy, Raynaud/telangiectasia or other, CK values (U/l) and the manual muscle testing (MMT8) score, if available.

### Statistical analyses

Descriptive statistics (mean, standard deviation (SD) and percentages) were used to summarize patients’ characteristics, treatments and outcomes for each calendar year. As the NDB exists for more than two decades, several variables have been added at certain time points. If applicable, the year in which a new variable was added is reported. To account for a case mix bias regarding disease duration, physician-reported disease activity and patient-reported outcomes are displayed by disease duration categories (< 5 years, 5–10 years, > 10 years).

To test whether outcomes have improved over time, patient-reported outcomes were compared with the Cochrane–Armitage test for trend.

From the supplementary questionnaire, the frequencies of phenotypes, diagnostics, organ involvement and autoantibodies are reported. Patients’ characteristics, comorbidities and patient-reported outcomes are reported for PM, DM, ASS and overlap-myositis. Patients were not reclassified if myositis-specific antibodies, e.g., Anti-Jo-1, were positive.

Ethical approval was obtained from the Ethics Committee of the Charité – University Medicine Berlin (EA1/196/06) in February 2007. This research was conducted in agreement with the Declaration of Helsinki.

## Results

### Cross-sectional trends

#### Characteristics

From 1997 to 2017, a total of 1079 patients (2355 visits) with IIM were documented in the NDB. Annually, the visits of 34 (in 2005) to 187 patients (in 2000) were recorded. Since 2005, the number of participating institutions and consequently the number of patients have decreased due to a switch to electronic documentation. Between 76% (1997) and 60% (2015) of the patients were female without a trend in the proportion over the years. Over the period of 20 years, the mean age increased from 52 to 58 years. The proportion of patients with long disease duration also increased over the years (Table 1).

**Table 1:** Patient characteristics 1997–2017

		1997	1999	2001	2003	2007	2009	2011	2013	2015	2017
*N*		*170*	*174*	*175*	*161*	*64*	*81*	*94*	*82*	*83*	*72*
Women, %		76	72	76	73	63	62	65	66	60	72
Age (years), mean		52	53	53	52	55	56	55	57	57	58
Age at disease onset (years), mean		46	46	46	44	46	47	47	48	47	48
Symptom duration (years), %	≤ 2 years	30	28	24	25	25	21	15	15	5	9
	2–5 years	29	32	28	26	20	23	25	19	30	17
	5–10 years	20	23	29	22	25	20	33	39	30	34
	> 10 years	21	17	20	27	29	37	27	28	34	40
*Medication*											
Nonselective NSAIDs, %		13	12	9	7	18	18	19	22	16	13
Glucocorticoids, %		84	82	82	71	78	81	78	73	66	58
of those	< 7.5 mg	54	56	54	59	60	71	80	79	88	83
Azathioprine, %		39	36	40	41	22	34	33	31	32	26
Methotrexate, %		20	26	29	20	29	21	33	35	34	32
Cyclosporine A, %		3	2	2	5	16	16	12	10	9	7
Cyclophosphamide, %		7	10	13	8	2	1	1		3	3
Antimalarials, %		9	7	4	7	4	6	5	6	7	4
Mycophenolate mofetil, %	Since 2005					4	9	9	6	8	8
TNF inhibitors, %	Since 2000			0	0	0	0	0	0	4	1
Rituximab, %	Since 2006					2	4	5	7	7	13

*NSAIDs* nonsteroidal antirheumatic drugs, *TNF* tumor necrosis factor

Due to a small case number the year 2005 is omitted

#### Treatments

Glucocorticoids were used less frequently in the more recent years (58% vs. 84%), *p* < 0.001. Glucocorticoid doses ≥ 7.5 mg were also used significantly less often (17% vs. 46%, *p* < 0.001). Azathioprine has been used less often in the recent years (26%), while methotrexate was used slightly more commonly (32%). Rituximab is used since 2006, and in 2017, 13% of the patients received rituximab. All immunosuppressive treatments are reported in Table 1. The prescription of physiotherapy was reported in 20%–34% of the patients, without a trend over the years.

### Physician- and patient-reported outcomes

Physicians have rated disease activity in recent years much lower than 20 years ago. In 2017, 94% of the patients reached low disease activity according to the physician assessment.

Over the years, the percentages of patients with favorable ratings on patient-reported outcomes increased significantly. The outcome gradings “low”, “good” and “little” were defined as  ≤ 3 on a 0–10 NRS. In 2017, 75% of the patients reported to have low pain compared to 47% in 1997. A good global health was reported by 51% of patients vs. 34% in 2002, little fatigue by 63% vs. 43% in 2007 and little sleeping disorders by 60% vs. 41% in 2007 (Table 2). No or little limitations in activities of daily living applied to 63% vs. 44% in 2007, and no anxiety/depression was reported by 75% vs. 53% in 2007, all *p* < 0.05. Details on outcomes are reported in Table 2.

**Table 2:** Physician and patient-reported outcomes 1997–2017, all %

	1997	1999	2001	2003	2007	2009	2011	2013	2015	2017
Physician-reported										
Disease activity										
Low (0–3)	59	58	64	65	76	82	77	87	91	94
Moderate (4–6)	33	36	26	28	20	15	19	10	7	6
Severe (7–10)	8	6	9	7	4	3	4	3	1	
Patient-reported										
Physical function										
Low limitation (> 70)	53	59	53	68	65	65	65	66	61	66
Limited (50–70)	26	19	24	11	10	13	12	9	20	16
In need of help (≤ 50)	22	22	22	21	25	22	23	25	20	18
Pain										
Low (0–3)	47	50	51	50	46	52	58	58	55	75
Moderate (4–6)	34	27	30	32	36	32	22	24	30	17
Severe (7–10)	19	23	19	18	18	17	20	19	15	8
Global health, since 2000										
Good (0–3)			35	36	36	32	43	37	42	51
Moderate (4–6)			39	43	45	55	35	46	26	37
Poor (7–10)			26	21	20	13	22	17	32	12
Fatigue, since 2007										
Little (0–3)					43	46	43	48	50	63
Moderate (4–6)					37	31	33	23	31	19
Severe (7–10)					20	24	25	28	19	17
Sleeping disorder, since 2007										
Little (0–3)					41	57	41	62	52	60
Moderate (4–6)					37	25	33	22	29	21
Severe (7–10)					22	18	26	17	19	19
Anxiety/depression, since 2007										
No					53	60	66	65	72	75
Moderate					43	40	29	34	24	25
Severe					4		4	2	4	

Outcomes are reported on numeric rating scales from 0 to 10 [14]. Physical function is reported from 0–100, 100 representing full function [16]

**Fig. 1:**
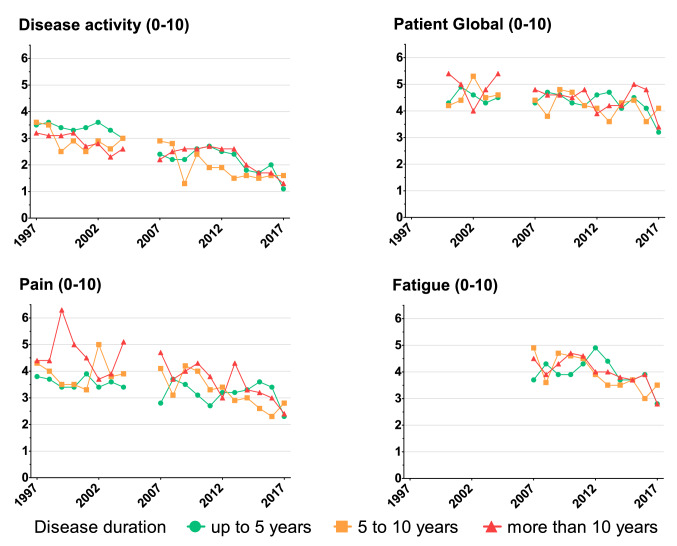
Trends in outcomes in inflammatory myopathies 1997–2017. Displayed are mean values of numeric ratings from 0 to 10. Disease activity is reported by the rheumatologist (0: no activity to 10: highest activity). Patient global, pain and fatigue are reported by the patients (0: no to 10 worst). The years 2005 and 2006 are omitted due to case numbers < 50

Improved patient-reported outcomes and physician-reported disease activity were observed in all disease duration categories (Fig. 1).

### Hospitalization and work participation

Fewer patients were hospitalized due to IIM in 2017 (18%) compared to 1997 (34%). Employment of patients < 65 years was 53% in both 1997 and 2017. Among employed patients, sick leave due to IIM during the last 12 months decreased from 52% in 1997 to 24% in 2017. The proportion of patients with early retirement decreased from 23 to 9% (patients ≤ 65 years) (Table 3).

**Table 3:** Hospitalization and work participation 1997–2017

	1997	1999	2001	2003	2007	2009	2011	2013	2015	2017
Hospitalized due to IIM in the past year, %	34	38	32	26	32	17	19	13	15	18
*Persons* < *65 years old, N*	*142*	*131*	*138*	*123*	*47*	*57*	*65*	*54*	*57*	*46*
Employed (< 65 years old), %	53	45	39	39	44	38	47	47	53	53
Employed part time among employed, %	–				12					24
Sick leave due to IIM in the past year among employed, %	52	26	46	44	50	38	50	27	26	24
Early retirement (< 65 years old), %	23	27	32	31	27	38	33	24	22	9

*IIM* Idiopathic inflammatory myopathies

### Comparison of subtypes from supplementary questionnaire

The supplementary questionnaire was available for 187 patients (Table 4). Physician-reported subtypes were PM (*n* = 82, 44%), DM (*n* = 62, 33%), ASS (*n* = 18, 10%), overlap (*n* = 15, 8%), inclusion body myositis (*n* = 3, 2%), necrotizing myositis (*n* = 1, 0.5%) or unspecific subtype (*n* = 5, 3%). In 11%, the patients were assigned to two phenotypes, mostly PM and overlap or PM and ASS. When diagnostic confirmation was performed, 82% showed CK/transaminase elevation, 66% myositis-specific antibodies, 87% muscle biopsy and 79% MRI findings. CK values differed considerably and were lowest in patients with DM and overlap. The MMT8 score was only obtained in 5 cases.

**Table 4:** Subanalysis: characteristics by physician-reported phenotypes

	Total	PM	DM	ASS	Overlap
*N*	*187*	*82*	*62*	*18*	*15*
*Characteristics*					
Sex, female n (%)	113 (61)	50 (61)	40 (65)	8 (44)	12 (80)
Age, mean (SD)	59 (14)	61 (13)	58 (14)	57 (14)	51 (18)
*Autoantibodies n (%), total n* = *131*					
Any autoantibodies	86 (66)	41 (71)	15 (41)	17 (94)	11 (79)
Of those Anti-Jo-1	49(57)	23(56)	5(33)	16(94)	4(36)
Mi2	6(7)	2(5)	4(27)	*0 (0)*	*0 (0)*
SRP	4(5)	4(10)	0 *(0)*	*0 (0)*	*0 (0)*
*Organ manifestation n (%), total n* = *186*					
Muscular	157 (84)	71 (87)	51 (82)	14 (78)	13 (87)
Skin	59 (32)	3 (4)	45 (73)	3 (17)	7 (47)
Arthritis	45 (24)	20 (24)	9 (15)	10 (56)	6 (40)
Interstitial lung disease	46 (25)	13 (16)	12 (19)	15 (83)	4 (27)
Dysphagia	26 (14)	12 (15)	11 (18)	0 (0)	2 (13)
Raynaud/telangiectasia	18 (10)	5 (6)	4 (7)	3 (17)	6 (40)
Cardiomyopathy	15 (8)	5 (6)	6 (10)	1 (6)	2 (13)
Pulmonary hypertension	5 (3)	0 (0)	4 (7)	1 (6)	0 (0)
*Comorbidities, n (%), total n* = *157*					
Hypertension	71 (45)	34 (49)	26 (46)	4 (24)	7 (54)
Coronary heart disease	33 (22)	19 (26)	11 (19)	2 (12)	1 (8)
Osteoporosis	30 (19)	14 (20)	11 (19)	2 (12)	3 (23)
Osteoarthritis	32 (21)	16 (23)	10 (18)	5 (29)	1 (8)
Diabetes	25 (15)	15 (21)	8 (14)	1 (6)	1 (8)
Renal disease	19 (12)	9 (13)	6 (11)	3 (18)	1 (8)
Depression	13 (8)	6 (9)	5 (9)	1 (6)	1 (8)
Malignant neoplasm	13 (8)	7 (10)	5 (9)	0 (0)	1 (0)

*PM* Polymyositis, *DM* dermatomyositis, *ASS* anti-synthetase syndrome

Due to the small number of cases, the other phenotypes are not listed

Of 131 patients with known autoantibody status, 66% had positive autoantibodies: Anti-Jo-1 (57%, *n* = 49), anti-Mi-2 (7%, *n* = 6), anti-SRP (5%, *n* = 4) and other (documented as ANA, ENA, SSA, RNP, PM-Scl, PL-7, proteasome, RO, Scl-70: 27%, *n* = 35). Anti-Jo-1 was present in all physician-reported subtypes: PM (56%, *n* = 23), DM (33%, *n* = 5), overlap (36%, *n* = 4) and ASS (94%, *n* = 16).

While muscular involvement was frequent across all subtypes (78–87%), other organ manifestations varied considerably. ILD (83%) and arthritis (56%) were predominant in ASS, skin affection (73%) in DM, Raynaud (40%) and cardiomyopathy (13%) in overlap-myositis. If any myositis-specific autoantibodies, especially Anti-Jo-1 antibodies, were present, patients had more frequently interstitial lung disease (61% vs. 17%, *p* < 0.001) and arthritis (43% vs. 16%, *p* < 0.001), but had less often skin involvement (12% vs. 42%, *p* < 0.001).

While hypertension and osteoporosis were most frequent in overlap-myositis, malignant neoplasms were found in 9% of patients with DM and osteoarthritis (29%) was more common in ASS.

**Fig. 2:**
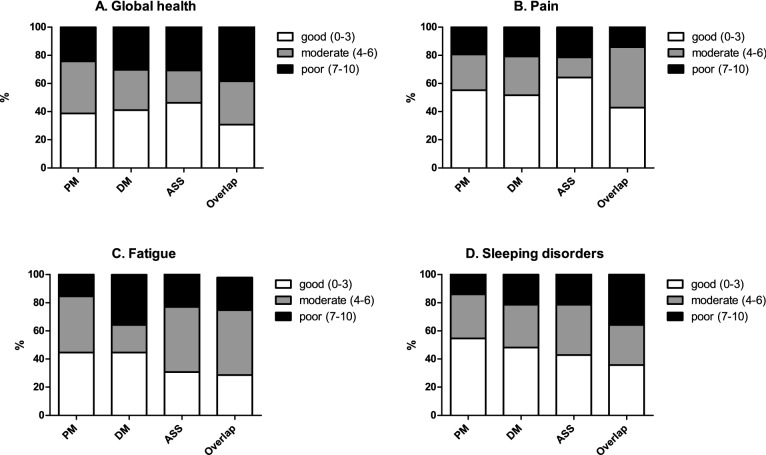
Patient-reported outcomes in idiopathic inflammatory myopathies. *PM* Polymyositis, *DM* dermatomyositis, *ASS* anti-synthetase syndrome, overlap overlapping-myositis, all classified by the rheumatologist. All outcomes were assessed on numeric rating scales (0–10)

In those 186 patients, the most frequently self-reported symptoms were limitations in global health (60%), fatigue (57%) and sleeping disorders (51%), and all of them were most frequent in overlap-myositis (Fig. 2). Patients with pulmonary hypertension (*n* = 5) and cardiomyopathy (*n* = 15) showed poor cross-sectional outcomes on global health, physical activities and fatigue [18].

## Discussion

With a total of 1079 patients, data from a comparatively large number of patients with IIM have been collected in the NDB over the past 20 years. Overall, a trend toward a better disease activity control with reduced needs of glucocorticoids was observed. Patient-reported outcomes on disease burden, work ability and hospitalization also improved. In 2017, 1 of 8 patients received rituximab and a further increase in more specific and effective therapies can be expected in future. Besides rituximab, baricitinib, triple regimens and other new treatment options bear the potential to improve the situation of patients with IIM [19].

Immunosuppressive treatment was less frequently used compared to the European registry [8]. Irrespective of the myositis phenotype, mean glucocorticoid doses remained above 7.5 mg prednisone equivalent daily in a relevant number of patients, indicating the need for more efficacious treatments. A Cochrane review reports a lack of high-quality RCTs that assess the efficacy and toxicity of immunosuppressants in inflammatory myositis [20]. So far, methotrexate and azathioprine showed similar survival rates as first-line immunosuppressives in a retrospective cohort [21] and they are also the most frequently used substances in our database.

As expected, organ involvement followed the diagnostic classification with ILD and arthritis associated with ASS and skin manifestation associated with DM. Patients suffering from cardiac manifestation and pulmonary hypertension frequently reported poor outcomes on all PRO dimensions. The dimensions most frequently reported by all myositis patients were fatigue and limitations in global health, while severe pain was reported by one-fifth. A systematic review on health-related quality of life in myositis patients revealed the need for further studies because existing data were scarce and heterogeneous [2]. Our data support a very heterogeneous picture of myositis patients, making it difficult to obtain robust results referring to small patient groups.

*Limitations and Strengths.* We report cross-sectional data, so that no causal relationship can be drawn regarding the source of outcome impairments. Organ involvement was reported as ever and may be treated sufficiently, so that it does not interfere with PROs anymore. Patient numbers on ASS, overlap, inclusion body myositis and necrotizing myositis were low, and these are not suitable to acquire robust data. As the NDB covers a wide range of inflammatory rheumatic diseases, specific data on IIM are lacking. The 2017 EULAR classification criteria cannot be applied [22]. The retrospective collection of additional myositis-specific information was a way to counter this limitation but is not a sufficient substitute for prospective data collection. Today, numerous disease-specific standardized and validated core set measures such as the Myositis Activities Profile (MAP) are available [23]. However, they are not used in everyday clinical practice. The NDB illustrates the routine rheumatological care in rheumatic centers in Germany. The Manual Muscle Testing (MMT) was collected only in 3% of IIM patients. Therefore, cross-disease measuring tools may be an appropriate tool for mapping changes over time in patients from routine care. The strength of the NDB is the long-term monitoring of PROs, enabling to provide valid data on the impairment regarding fatigue, pain and other general symptoms in a sufficient number of myositis patients. With pain, fatigue and sleeping disorders, three of the patient-prioritized domains [10] are available for a long observation period. However, muscle symptoms as the main domain in the OMERACT core set [11] are not reported. Recent advances in the outcome assessment of myositis including patient-reported outcome measures enable a more specific future outcome research in patients with IIM [24, 25].

We can conclude from our data that in the NDB, physician-reported disease activity and patient-reported disease burden in IIM have improved within the last 20 years. Fatigue, impaired global health and sleeping disorders are frequently reported domains in IIM that may be considered in future research.

## Data Availability

Availability of data and material: No additional data are available.
